# GRATITUDE WHILE DRINKING, GRATITUDE WHILE RECOVERING: A STUDY OF ALCOHOL USE DISORDERS

**DOI:** 10.31886/jors.13.2019.39

**Published:** 2019-03-08

**Authors:** AMY R. KRENTZMAN, MICHAEL T. M. FINN

**Affiliations:** UNIVERSITY OF MINNESOTA SCHOOL OF SOCIAL WORK, ST. PAUL, MINNESOTA, USA; OSHER CENTER FOR INTEGRATIVE MEDICINE AT VANDERBILT UNIVERSITY MEDICAL CENTER, NASHVILLE, TENNESSEE, USA

**Keywords:** gratitude, recovery, alcohol use disorder, controlled drinking

## Abstract

**Background.:**

For decades researchers have debated whether those diagnosed with alcohol use disorders can return to non-problematic drinking. Now, recovery researchers are measuring aspects of wellbeing in addition to aspects of pathology, producing surprising findings that have added to the debate. Recent studies show that some with alcohol use disorders who continue to drink endorse high levels of positive psychosocial functioning.

**Objectives.:**

Employ trait gratitude as a marker of wellness to answer the following questions: how do individuals who continue to drink but endorse high gratitude at follow-up differ from peers at baseline? Does trait gratitude correlate differently with demographic, psychosocial, and clinical factors for abstinent members of Alcoholics Anonymous (AA) versus actively drinking non-AAs?

**Methods.:**

275 individuals with alcohol dependence were assessed for trait gratitude at 2.5-3-year follow-up in a naturalistic, longitudinal study. The sample was assessed on psychosocial and clinical indicators at baseline and follow-up.

**Results.:**

Drinkers who endorsed high gratitude had higher socioeconomic status, greater levels of positive spirituality, more stable personality indicators, less addiction severity, fewer negative life events, and fewer psychiatric symptoms than their peers at baseline. For actively drinking non-AAs, trait gratitude correlated differently, and positively, with years of education, income, and purpose in life compared with sober AA members. For AA members, gratitude correlated with AA involvement and length of sobriety.

**Discussion.:**

Across multiple domains, a subset of drinkers report doing relatively well despite meeting criteria for alcohol dependence. Trait gratitude correlates differently with other constructs for AAs versus non-AAs, indicating that gratitude for recovery might be contextually sensitive, operating differently within and without the structure of AA.

## Introduction

Since 1962 scholars have debated whether it is possible for an individual with alcoholism to return to controlled drinking (Rosenberg, 1993). Over the past several decades, research has shown that at least some individuals who formerly had alcohol problems continue to drink but at lower levels (Helzer et al., 1985) and without concurrent problems (Ilgen, Wilbourne, Moos, & Moos, 2008). After reviewing the research, Rosenberg (1993) concluded that controlled drinking had been observed among individuals who had less severe dependence, were younger, were women, were employed, and were more stable psychologically and socially. However, there is also evidence that an unproblematic return to drinking is not as stable a solution as abstinence (Ilgen et al., 2008).

Work in this area is being viewed through a new lens based on shifts in the field that occurred over the past two decades. Researchers and clinicians have been moving away from an acute-care model to a chronic-care model of addiction, which is truer to the chronic nature of the illness (McLellan, Lewis, O’Brien, & Kleber, 2000). No longer are treatment centers, halfway houses, and 12-step programs the only offerings for individuals with addictions. Now a wide range of recovery-oriented systems of care are available. These recovery-oriented systems of care provide long-term support through active addiction, its resolution, and all stages of recovery (Kelly & White, 2011). Such recovery-oriented systems of care include free-standing social service agencies with a primary focus on recovery (Valentine, 2011), recovery-oriented high schools (Moberg & Finch, 2008; Vogel, 2009), recovery programs on college campuses (Bell et al., 2009), and a wider diversity of sober living residences, e.g., (Edwards et al., 2017; Mericle, Polcin, Hemberg, & Miles, 2017; Polcin, Korcha, Bond, Galloway, & Lapp, 2010).

With this shift in the view of addiction as chronic versus acute came acceptance of wider, more comprehensive, and more diverse definitions of recovery. No longer is abstinence the sole criterion. Recovery has been broadened to include markers of prosperity in other life domains. These domains include personal health and citizenship (Betty Ford Institute Consensus Panel, 2007, 2009), care for one’s self and others, personal development (Kaskutas et al., 2014), psychological health, physical health, education, employment, self-knowledge, and wellbeing (Neale et al., 2014).

Researchers are now interested in measuring aspects of wellbeing in addition to aspects of pathology in studies of alcohol use disorders and their resolution. One reasonably might expect that those who resolve their alcohol use disorders with abstinence would experience a sharp rise in wellbeing and psychosocial functioning and that those who continue to drink would exhibit poor indicators of wellbeing and psychosocial functioning. However, the study of wellbeing in recovery has produced some surprising findings that have converged with the debate over controlled drinking.

Witkiewitz and colleagues (2019) conducted a secondary analysis of Project MATCH to identify diverse recovery profiles three years after abstinence-based treatment. The majority of participants in Project MATCH met criteria for DSM IV alcohol dependence, the balance met criteria for DSM IV alcohol abuse (American Psychiatric Association, 1994; Krentzman, Cranford, & Robinson, 2013). The research team used latent profile analyses to determine classes, or groupings, of distinct patterns of recovery. The research team included diverse indicators of psychosocial functioning, as well as drinking behavior, to define the recovery profiles. The researchers found three classes that one might expect: frequent heavy drinkers reporting poor functioning (15.8% of the sample), infrequent heavy drinkers reporting poor functioning (16.1% of the sample), and abstinent and infrequent light drinkers reporting high functioning (51.2% of the sample). In addition, however, the research team identified a less-anticipated class: individuals who drank heavily on occasion but who nonetheless reported high psychosocial functioning (16.9% of the sample). The researchers pointed out that psychosocial functioning was based on participant self-report; it is unclear whether friends, family, and employers of occasional heavy drinkers would agree that the individual was functioning at a high level. However, the results are nonetheless surprising: some who had been diagnosed with alcohol use disorders who had been through abstinence-based treatment still drank heavily at times three years later but were otherwise doing well. They had low unemployment, low levels of depression and anxiety, low difficulty concentrating, little evidence of problematic social behaviors, and greater satisfaction with life.

A similar unexpected finding was reported in a study of trait gratitude among individuals diagnosed with DSM IV alcohol dependence who had attended abstinence-based treatment (Krentzman, 2017). Trait gratitude is the dispositional tendency to feel thankful for positive things in life; individuals with high trait gratitude feel thankful more frequently, more intensely, and via a greater number of prompts (Wood, Froh, & Geraghty, 2010). Krentzman’s study showed that some individuals who continued to drink frequently six months after treatment reported high levels of trait gratitude. Those who scored high affirmed that they “have so much in life to be thankful for,” are “grateful to a wide variety of people,” and are able to “appreciate the people, events, and situations” that have been part of their lives. This finding was particularly surprising because anecdotal and documentary evidence link gratitude to abstinence-based addiction recovery and 12-step programs such as Alcoholics Anonymous (AA) and Narcotics Anonymous (NA) (Chen, 2017; Krentzman, in press; LaBelle & Edelstein, 2018). Therefore, it was not expected that some who were diagnosed with alcohol use disorders would endorse high levels of trait gratitude while actively drinking. Furthermore, the study’s findings suggested that among those who drank frequently, the higher their trait gratitude, the less likely they were to change their drinking six months later. High gratitude was not associated with future changes in drinking. The Krentzman study employs a different subset of the same parent dataset as the current study.

## The Current Study

These surprising findings warrant further investigation. Therefore, we conducted a pair of studies that each answer one research question. Study 1 takes a closer look at the ways in which individuals with alcohol dependence who at follow-up are still drinking, but report high trait gratitude, differ from their peers at baseline. Specifically, Study 1 answers the question: how do high gratitude, actively drinking individuals differ from other individuals diagnosed with alcohol use disorders on a wide range of demographic, psychosocial, and clinical measures at baseline? The answer to this question will help us to better understand individuals who continue to drink but rate themselves highly on self-reported measures of wellbeing.

Study 2 compares the nature of trait gratitude endorsed by two groups of individuals with alcohol dependence: non-AA members who are still drinking and AA members sober at least 90 days. We chose these groupings intentionally to showcase contrasting experiences of gratitude. Aspects of AA and NA focus on fostering and practicing gratitude (Chen, 2017; Krentzman, in press; LaBelle & Edelstein, 2018); for example, the 10th step recommends recounting one’s blessings on a daily basis (AA World Services, 1953). Therefore, the gratitude endorsed by AA members should display a meaningful contrast to the gratitude endorsed by individuals who are drinking non-AA members. Study 2 addresses the following research question: does trait gratitude correlate differently with demographic, psychosocial, and clinical factors for individuals with alcohol dependence who are drinking non-AA members versus abstinent members of AA? The answer to this question will help us to better understand diverse experiences of gratitude among individuals with alcohol use disorders and whether trait gratitude experienced by drinking non-AA members is the same construct as trait gratitude experienced by sober AA members.

## Methods

### The Life Transitions Study (LTS).

We employed data from the LTS to address our research questions. The LTS is a longitudinal, descriptive study of 364 individuals diagnosed with DSM IV alcohol dependence who were followed prospectively for 2.5-3 years. Individuals were assessed in person at baseline and every six months on a wide range of clinical and psychosocial indicators. The study was conducted from 2004 to 2009 in the Midwestern United States and was originally designed to focus on the spirituality, religiousness, and alcohol use of a diverse sample of individuals with alcohol use disorders. The diversity of the sample was achieved by recruiting participants from different sources: n=157, 43.1%, came from a university-affiliated abstinence-based addiction treatment program; n=80, 22.0%, came from a US Department of Veterans Affairs abstinence-based addiction treatment program; n=34, 9.3%, came from a counseling center that permitted reduced drinking as a treatment goal; and 93, 25.5%, came from the local community comprising individuals not enrolled in treatment at baseline.

The current study makes maximum use of the final follow-up assessment of trait gratitude in the LTS. In the LTS, trait gratitude was assessed at baseline and at the six-month follow-up for only the first 67 participants (for an analysis of gratitude among these participants, see Krentzman, 2017). Gratitude was then dropped from the protocol. However, trait gratitude was assessed for all participants who completed the final follow-up 2.5-3 years after baseline with the exception of one person present at the final follow up but for whom there was missing gratitude data (for a total subsample of n=275, 75.5% of the baseline LTS sample). For Studies 1 and 2, we employed this subset of the LTS to answer our research questions.

Those included in our analyses and those excluded (because they did not attend the final follow-up interview) were similar on all demographic characteristics and on most clinical characteristics at baseline with a few exceptions. Those retained in the study had lower AA involvement (.9 versus 1.3, p<.05), lower average drinks per drinking day (9.0 versus 11.2, p<.05), and were less likely to want abstinence as a treatment outcome (69% versus 82%, p<.05).

### Participants.

On average at baseline, individuals in this subset of the LTS were 44.2 (SD=13.0) years of age and had 14.5 (SD=2.4) years of education. Average household income was $44,689 (SD=28,062). The majority were white (80.7%), 34.9% were female, 38.5% were married or cohabitating, and 57.8% were employed. At baseline on average, participants drank 9.0 (SD=7.6) drinks per day, were abstinent from alcohol 56.1% (SD=31.1%) of the past 90 days, had heavy drinking days 31.8% (SD=29.2%) of the past 90 days, and reported 25.1 (SD=27.5) days since their last drink. They reported average scores of 20.5 (SD=11.5) on a measure of drinking consequences, 23.3 (SD=12.9) psychiatric symptoms, and 2.2 (SD=1.8) negative life events experienced in the past six months. Most had a family history of alcohol problems (87.6%), 50.2% had had previous treatment for alcohol problems, 68.7% desired abstinence. The majority (57.1%) had severe alcohol dependence, 16.7 had moderate, and 26.2 had mild alcohol dependence at baseline. The average age of onset of alcohol dependence symptoms was 28.8 (SD=12.4) years of age.

### Recruitment.

Recruitment at the abstinence-based treatment programs began with the clinical records which were reviewed to identify individuals who appeared to meet study criteria. Research staff subsequently approached these prospective respondents. Recruitment at the site which permitted reduced drinking as a treatment goal began with staff who identified clients at their agency who appeared to meet study criteria and then connected these individuals to the study research staff. Individuals in the community were recruited with advertisements in the newspaper. Inclusion criteria included being 18 years of age or older, meeting criteria for alcohol dependence, recent drinking, and English literacy. Exclusion criteria included active suicidal ideation, homicidal ideation, and psychosis. The majority (78%) of those approached enrolled in the study. The appropriate institutional review boards approved this study. For more on the LTS, please see (Krentzman, Webb, Jester, & Harris, 2018; Robinson, Krentzman, Webb, & Brower, 2011).

### Measures

#### Gratitude.

Trait gratitude was assessed with The Gratitude Questionnaire-Six Item Form (McCullough, Emmons, & Tsang, 2002. The instrument uses six items and a 7-item Likert-Type response format ranging from 1=strongly disagree to 7=strongly agree. A sample item is, “If I had to list everything that I felt grateful for, it would be a very long list.”

#### Demographics.

Demographic variables included age, years of education, income, employment, gender, race, and marital status.

#### Psychosocial characteristics.

Psychosocial characteristics included dimensions of spirituality and personality, as follows:

##### Daily spirituality.

The Daily Spiritual Experiences scale measured experiences of spirituality as part of one’s everyday life (Underwood & Teresi, 2002). The measure contains 16 items that ask, on a 6-point Likert-type scale, how many times a day a person experiences the given aspect of spirituality. Questions include daily experiences of awe, a sense of connection to all of life, feeling God’s presence, and being thankful for blessings. When asked to answer survey questions in the LTS, respondents were invited to replace “God” with another word that “calls to mind the divine or holy” if they wished to.

#### Spiritual/religious practices.

This construct was measured by the Private Religious Practices measure (Fetzer Institute, 2003). This 5-item measure assesses the frequency of engaging in prayer and religious practices in informal and non-institutional settings.

#### Positive and negative religious coping.

These constructs were assessed using a custom measure that combined two versions of the Brief Religious Coping instrument (Fetzer Institute, 2003; Pargament, Smith, Koenig, & Perez, 1998). This measure assesses the individual’s response to/experience of God during stressful times; specifically, the degree to which God is experienced as benevolent (positive) and the degree to which God is experienced as punitive (negative). One item was removed from the original measure to be inclusive of those who may not attend church at all. See Krentzman et al. (2017) for a more detailed description of the measure construction process.

#### Purpose in life.

This construct was measured by the Purpose in Life Scale (Crumbaugh, 1968; Crumbaugh & Maholick, 1964). This measure uses 20 items to assess overall orientation of a feeling of purpose. It gives a brief prompt, followed by a seven-point scale with anchors at either end; point 4 is labeled neutral. For example, one item reads “My life is…”, and is given the following three anchors: 1: filled only with despair, 4: neutral, 7: running over with exciting good things.

#### Forgiveness of self and others.

These constructs were measured by two subscales of the Behavior Assessment System (Mauger et al., 1992). Each subscale has 15 true/false items that tap forgiveness behaviors. The forgiveness of others subscale involves holding grudges, being punishing toward others, among other related items. The forgiveness of self-subscale involves considering oneself as sinful, tendency to find oneself guilty, to self-condemn, among other related items. The subscales were reverse-coded to represent presence of forgiveness behaviors.

#### Personality.

The NEO Five Factor Inventory is a widely used measure of the five-factor approach to personality (Costa & McCrae, 1992). As such, it assesses neuroticism, extraversion, agreeableness, openness, and conscientiousness, each on a continuum. It is composed of 60 items, 12 per each personality factor, that give self-descriptive statements written in the first-person and ask the respondent to rate their degree of agreement on a 5-point Likert-style scale. According to Goldberg (1990), the NEO factors can be described as follows: higher neuroticism indicates more nervousness, moodiness, and temperamentality; higher extraversion indicates more talkativeness, assertiveness, and activity level; higher conscientiousness indicates more organization, thoroughness, and reliability; higher agreeableness indicates more kindness, trust, warmth; and higher openness indicates more imagination, curiosity, and creativity. Previous research on the LTS sample showed the relevance of personality factors, particularly neuroticism, in predicting time to relapse when controlling for predictive clinical and demographic factors (Finn & Robinson, 2012).

#### Clinical characteristics.

Clinical characteristics assessed alcohol use, mental health, addiction history, severity of alcohol use disorder, recovery behaviors and goals, and type of treatment at baseline, as follows.

#### AA involvement.

This construct was measured using 5 original items from the AA Involvement Scale (Tonigan, Connors, & Miller, 1996): attending 90 meetings in 90 days, identifying as an AA member, having a sponsor, being a sponsor, and celebrating an AA anniversary. Participants responded to each item yes or no, with each yes response adding one point to the total.

#### Drinking consequences.

This construct was measured by the SIP instrument (Miller, Tonigan, & Longabaugh, 1995). The SIP instrument uses 15 items and a 4-point Likert-type response format ranging from 0=never to 3=daily or almost daily. A sample items is, “I have been unhappy because of my drinking.”

#### Number of psychiatric symptoms.

This construct was measured with the Brief Symptom Inventory (Derogatis & Melisaratos, 1983). The instrument assesses presence or absence of 53 psychiatric symptoms that have been bothersome in the past week, e.g., feeling “nervousness or shakiness inside.” The Positive Symptom Total scale, derived from this instrument, was used in the current study. This scale is a total count of any symptoms endorsed.

#### Negative life events experienced in the past 6 months.

This construct was assessed with the brief life events questionnaire (Brugha & Cragg, 1990). This questionnaire lists 12 negative life events and asks respondents to indicate whether they have experienced them in the last six months. A sample item is, “you yourself suffered a serious injury, or an assault.” Participants endorse yes or no for each item and each yes answer earned 1 point toward a total scale score.

#### Age at first symptoms of alcohol use disorder.

This was measured with a single item asking respondents how old they were when they first exhibited the symptoms of alcohol use disorders that they had reported.

#### Wanting abstinence as a treatment outcome.

This construct was measured by a single item: “How about being abstinent from alcohol? Is that something you want to do?” to which respondents answered yes, no, maybe, or don’t know. Maybe and don’t know were re-coded as no.

#### Having had previous treatment.

This construct was measured by a single item: “Have you ever been in treatment before for your alcohol problem?” to which respondents answered yes or no.

#### Family history of alcohol problems.

This construct was measured by a single item: “Has anyone in your family had problems with alcohol?” to which respondents answered yes or no.

#### Dependence severity.

This construct was measured by the Structured Clinical Interview for the DSM IV (First, Spitzer, Gibbon, & Williams, 1997) which allows for identification of alcohol dependence symptoms and categorization of respondents into three severity categories: mild (3-4 symptoms), moderate (5 symptoms), and severe (6-7 symptoms). We also employed the Structured Clinical Interview to indicate whether symptoms of physiological dependence were present, that is, symptoms of either tolerance or withdrawal.

#### Alcohol use.

The Time-Line Follow-Back instrument was used to assess alcohol use (Sobell, Brown, Leo, & Sobell, 1996; Sobell & Sobell, 1992). This calendar-based method asks participants to identify national and religious holidays and other notable life events that occurred in the past 90 days. The identified events serve as aids to memory, helping the respondent identify days when drinking occurred and how much was drunk on each drinking day. A standardized drink is used as a unit of measure. From this calendar method it was possible to calculate the percentage of days the participant was abstinent in the past 90 days, the average drinks per drinking day in the past 90 days, and the percent of heavy drinking days in the past 90 days. The instrument also produced a count of number of days since last drink.

#### Treatment type at baseline.

Individuals were coded as being in abstinence-based treatment at baseline if they were enrolled in the VA or University treatment programs. Individuals enrolled in the treatment program which permitted reduced drinking as a goal were indicated as such. The balance of the sample were not in treatment at baseline.

### Data Collection and Analyses

#### Study 1.

The purpose of Study 1 was to determine baseline differences between individuals who at final follow-up endorsed high trait gratitude while actively drinking (n=64) and their peers who also participated in the final interview in the study (n=211). We defined high trait gratitude as a score at or above the median of 36 in the final assessment of the Gratitude Questionnaire. We determined days since last drink using the time-line follow-up instrument (Sobell et al., 1996; Sobell & Sobell, 1992). Ongoing drinking was defined as having had a drink less than 14 days before. Previous research has indicated that 14 days of abstinence is a reasonable indication that a period of sobriety has begun; when a last drink is fewer than 14 days ago, it reasonably indicates ongoing drinking which can include some naturally occurring days of abstinence (Stout, 2000). We assessed for general differences in spirituality and personality between the two groups across a series of measures using two MANOVAs. We corrected for multiple comparisons at this level by setting each alpha at .025 (.05/2).

The next set of analyses are descriptive and exploratory, therefore, we did not correct for multiple tests. Differences between the two groups in the level of each individual measure of demographic, psychosocial, and clinical variables at baseline were determined by t-test analyses for continuous variables and Chi Square analyses for categorical variables.

#### Study 2.

The purpose of Study 2 was to compare how trait gratitude correlated with other demographic, psychosocial, and clinical variables for two groups categorized based on their status at the LTS final assessment interview: non-AA members who drank less than 14 days ago (indicating active drinking versus abstinence, Stout, 2000) and AA members who reported at least 90 days of continuous sobriety. We selected these group parameters to depict a strong contrast between active drinking behavior and sobriety in AA. We identified individuals who were or were not AA members based on their answer of “yes” or “no” to an item derived from the AA Involvement Scale (Tonigan et al., 1996): “Do you currently consider yourself to be a member of AA?” We assessed days since last drink using the time-line follow-up instrument (Sobell et al., 1996; Sobell & Sobell, 1992). We identified n=108 individuals at the final assessment interview who endorsed that they were not AA members and whose last drink was less than 14 days before the final interview. Days since last drink for this group ranged from 0-12. We identified n=55 individuals at the final assessment interview who endorsed that they were members of AA and also reported that their time since last drink was a minimum of 90 days. Days since last drink for this group ranged from 94-1184. Pearson correlations were conducted to test the relationship between trait gratitude and other continuous demographic, psychosocial, and clinical variables. We used a Fisher r-to-z transformation to compare the two groups on differences in correlation magnitude between gratitude and other factors.

## Results

### Study 1.

We began the analysis with two one-way MANOVA tests to investigate general differences at baseline in both spirituality and personality among the high gratitude drinking individuals when compared to others. We found a significant medium effect of differences between these groups across the measures of spirituality, F(7, 260) = 6.33, p < .001, eta2 = .145. For the measures of personality, we found a similar effect, F(5, 268) = 9.47, p < .001, eta2 = .15. Correcting for multiple comparisons, both findings remain significant.

Following, we explored specific measure-level differences. See Table 1 for a full report of these exploratory analyses. For spirituality measures, it appeared that the high gratitude drinking group had less negative religious coping, t(269) = −2.21, p = .03, more forgiveness of self, t(273) = 5.17, p < .001, and more purpose in life, t(270) = 5.56, p < .001. Regarding personality factors, we observed that all five NEO-FFI factors were significantly different, ps < .001. The consistency and magnitude of these differences was surprising; all gave the impression of more adaptive personality styles, particularly lower Neuroticism, among the high gratitude drinking group.

Likewise, across almost all clinical and demographic variables that we examined, the high gratitude, actively drinking group consistently had indicators of greater socioeconomic stability and health. They were younger with higher levels of education, had a higher average income, were more likely to be employed, and more likely to be married or cohabitating, ps < .05. They were also more likely to be female, p < .05.

The high gratitude, actively drinking group had fewer days abstinent over the past 90 days than others in the study and they drank less per day on average, ps < .05; they drank more frequently but less intensely. They had a higher rate of mild alcohol dependence and a lower rate of severe alcohol dependence, ps < .001. They had fewer psychiatric symptoms, p < .01, fewer negative life events, p < .05, and fewer drinking consequences, p < .001. Centrally, they had a much lower rate of a desire for abstinence, p < .001, a lower rate of having sought previous addiction treatment, p < .01, and were less likely to have considered themselves to be involved in AA, p < .001. They were less likely to be in abstinence-based treatment at baseline, p < .001. However, they were equivalent to their peers on a couple of important factors. They reported an equivalent rate of physiological dependence and reported a similar rate of heavy drinking days.

### Study 2.

**Figure 1.**
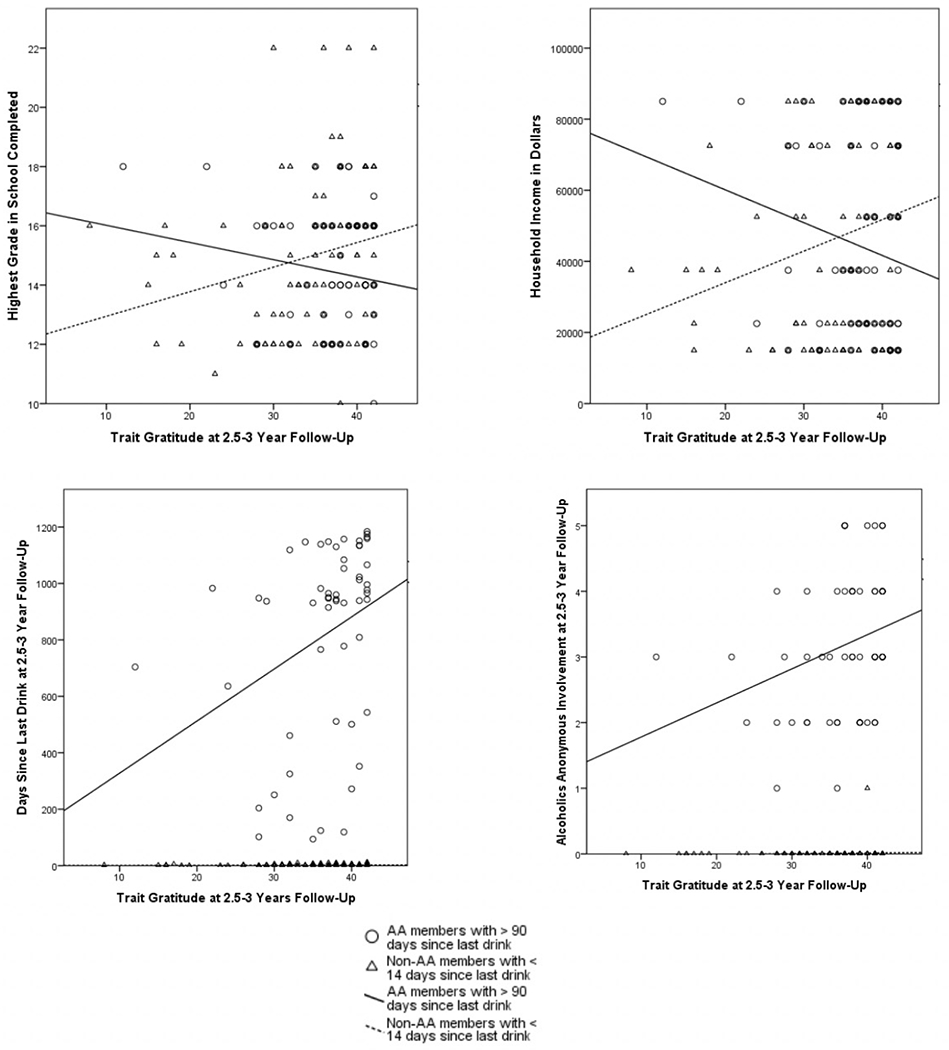
Correlations between trait gratitude and education, income, days since last drink, and Alcoholics Anonymous involvement as they vary by drinking behavior and Alcoholics Anonymous membership

We then sought to explore the role of AA involvement in how gratitude operates with personality, spirituality, and drinking variables. Thus, we explored the correlations of these variables within two groups: actively drinking non-AA members and AA members abstinent at least 90 days. See Table 2 for these results. These analyses were exploratory, intended to begin to understand how the context of AA may alter the influence of gratitude in a person’s life. Interestingly, years of education and income had low but significant positive correlations with high gratitude for actively drinking non-AA members, p < .05. Among those in AA with at least 90 days abstinent, there were no significant correlations between these factors – if anything, trending to a negative correlation (See Figure 1). Using a Fisher r-to-z transformation to compare the two groups, we found that there was a significant difference in correlation of gratitude with years of education, z = 2.35, p = 0.02; and with income, z = 2.47, p = 0.01 (see Figure 1). It appeared that gratitude played more of a role in days since last drink among AA members than actively drinking non-AA members, though this was not a detectable difference in correlation magnitude, z = −1.4, p = .16. Gratitude correlated significantly and positively with AA involvement for AA members, p < .05 (see Figure 1).

Purpose in life was highly correlated with gratitude among the non-AA group, r(106) = .64, p < .001, while it was moderately correlated among the AA group, r(53) = .37, p < .01. There was a significant difference in magnitude of these two correlations, z = 2.15, p < .032. This suggested that purpose in life tends to be more coincident with gratitude for persons outside of of AA than those within AA. There were less pronounced differences in the relation between gratitude and personality between these two groups. There was a marginal difference in the degree that openness co-occurred with gratitude, z = 1.75, p = .08, which had a small positive correlation in the non-AA group, r(106) = .24, p < .05 and no correlation in the AA group, r(53) = −.05, p > .90.

## Discussion

The current study extends what is known about the diversity of experiences individuals diagnosed with alcohol use disorders have with gratitude and its associations with drinking, abstinence, psychosocial characteristics, and clinical factors. Individuals with higher than median gratitude who were drinking at the final assessment of a 2.5-3-year longitudinal study, when compared to peers at baseline, were significantly better off on a wide range of demographic, spirituality, personality, and mental health characteristics. They had greater socioeconomic status and more stable social indicators. On some dimensions of spirituality, their scores indicated greater positive spirituality. They were less interested in abstinence and less likely to be abstinent. They reported low, if any, AA involvement. They drank more frequently but less intensely. Their diagnostic severity status was more likely to be mild than severe. They suffered less from drinking consequences, negative life events, and psychiatric symptoms. They were more likely to be female. These findings resonate with previous research on non-problem drinking among individuals with alcohol use disorders. Previous work has reported that those with the ability to moderate their drinking had less severe addiction histories and were higher functioning on a range of psychosocial indicators (Helzer et al., 1985; Ilgen et al., 2008; Rosenberg, 1993; Witkiewitz et al., 2019). As in the current study, several other studies found that this pattern of drinking was more achievable for women (Helzer et al., 1985; Ilgen et al., 2008; Rosenberg, 1993).

In Study 2, gratitude correlated differently with a range of demographic, psychosocial, and clinical factors for actively drinking non-AA members compared with AA members with 90 days or more of abstinence. Gratitude for the drinking non-AA members was more related to socioeconomic status, e.g., years of education and income, higher purpose in life, and a trend toward the personality dimension of openness. Not surprisingly, for AA members, gratitude was significantly and positively associated with days since last drink and AA involvement; this was not relevant for drinking non-AA members.

Given the widespread differences in NEO-FFI factor scores between high gratitude actively drinking persons and others, there may be a common factor accounting for much of these differences. In recent personality research, the role of demoralization, closely related to hope, as a separable factor from many personality scales has received much attention (Ben-Porath, 2012; Noordhof, Sellbom, Eigenhuis, & Kamphuis, 2015). This is in line with the hypothesis that demoralization is a pervasive factor across psychopathology and is the main reason that individuals seek out mental health services (Frank & Frank, 1993). Demoralization has been shown to account for much of the previously presumed differences in personality occurring in psychotherapy for depression (Noordhof, Kamphuis, Sellbom, Eigenhuis, & Bagby, 2018). This could be a major factor accounting for the widespread personality differences between these two groups: that the individuals in the high gratitude, actively drinking group were less demoralized in their lives.

### Limitations

We used secondary data to answer these research questions which limited our research design options. Trait gratitude was assessed for all study participants only at the 2.5-3 year follow-up. Therefore, we could not examine differences between groups in gratitude at baseline or longitudinally over time. Some have criticized the parceling of those who completely abstain from drinking from those who drink at low levels in alcohol use disorders research. Empirical evidence has shown that non-drinkers and low-risk drinkers look similar to one another on a range of factors (Witkiewitz et al., 2019). However, our decision to compare abstainers to drinkers was based on our desire to compare groups that were as different as possible (i.e., drinking non-AA members and abstinent AA members) to depict strong contrasts in this exploratory study. Individuals analyzed in this subset of the LTS completed the final assessment interview 2.5-3 years after baseline. They differed from respondents who did not participate in the final interview: at baseline they drank less intensely, were less likely to want abstinence, and were less involved in AA. In addition, the sample was primarily white and relatively highly educated. Therefore, generalization to all with alcohol use disorders should be undertaken with caution. This study was conducted among individuals diagnosed with alcohol dependence according to the DSM IV standards. The DSM V is in use at the time of this writing and conceptualizes the disorder along a continuum versus a clear cut point between abuse and dependence. It is not clear whether those in this study who drank but had high gratitude might have been misdiagnosed based on limitations in the DSM IV. Further improvement to the DSM criteria over time should improve accuracy when using this diagnostic tool.

### Future Directions

#### What do these findings tell us about gratitude?

The results of this study are in accordance with previous research which has shown that trait gratitude correlates positively with positive markers of wellbeing and negatively with negative markers of wellbeing. For example, trait gratitude has been shown to correlate positively with post traumatic growth (Kashdan, Uswatte, & Julian, 2006; Lies, Mellor, & Hong, 2014), positive mood (McCullough et al., 2002), and approach coping (Wood, Joseph, & Linley, 2007). Among AA and NA members, trait gratitude has been shown to correlate positively with length of sobriety, support from in recovery, step work, and AA promises coming true (LaBelle & Edelstein, 2018).

In previous research, trait gratitude has correlated negatively with psychopathology and substance misuse (Kendler et al., 2003), avoidant coping (Wood et al., 2007), negative affect (McCullough et al., 2002), and employee burnout (Chan, 2011; Kaplan et al., 2014). Among AA and NA members, trait gratitude was shown to correlate negatively with measures of stress and reported number of physical health symptoms (LaBelle & Edelstein, 2018).

In the current study, for both drinking non-AA members and for sober AA members, trait gratitude correlated positively with daily spiritual experiences, positive religious coping, purpose in life, forgiveness of others, forgiveness of self, and extraversion. Trait gratitude correlated negatively with neuroticism and the number of reported psychiatric symptoms.

The results of this study are the first empirical findings to suggest that the gratitude experienced during AA-mediated recovery might be qualitatively different than general trait gratitude, perhaps tapping a related but a different construct. This we conclude because of the different ways in which trait gratitude correlated with other aspects of wellness and pathology for AA compared with non-AA members. This provides evidence, which, pending replication, could signal a need to develop a new measurement instrument to assess gratitude for recovery distinct from trait gratitude. In general, the empirical research on gratitude in recovery is in its infancy. More work should be done to determine the ways in which gratitude is experienced in early, middle, and long-term recovery and the way that it changes over time. The development of an instrument to assess gratitude for recovery can be useful to these investigations.

#### What do these findings tell us about alcohol use disorders?

Some who are doing well on a wide range of socioeconomic and other psychosocial factors at baseline continue to drink and report high levels of gratitude at a 2.5 - 3-year follow-up. This study replicates what others have reported (Helzer et al., 1985; Ilgen et al., 2008; Rosenberg, 1993; Witkiewitz et al., 2019): low addiction severity is associated with positive psychosocial wellbeing even if still drinking. These results prompt a range of further questions: Do high-functioning individuals who continue to drink feel that their alcohol use disorder is “resolved”? Do they consider themselves “in recovery”? What would we learn if we followed such individuals over time? Would their drinking behavior be stable over time or unsustainable? Ilgen and colleagues (2008) found that less than half of those with alcohol use disorders who reported drinking without problems continued to report no problems with drinking at follow-up. In their study, abstinence was more strongly associated with longer-term stable remission than problem-free drinking was.

The results of the current study suggest that at treatment entry, those with low addiction severity and other favorable markers of psychosocial functioning might do well with a moderated drinking resolution to their alcohol use disorder. However, several cautions attend this suggestion. First, wellbeing indicators in this body of work have been collected via self-report. Collateral input, that is, information on participant wellbeing collected from friends and family, is necessary to gain a fuller, more accurate picture of positive functioning. Second, existing research suggests that good outcomes are less stable with moderated drinking compared with abstinence (Ilgen et al., 2008). Third, individuals with alcohol use disorders who continue to drink but report high levels of wellbeing are in the minority; they comprise a subset of larger samples of individuals with alcohol use disorders (Helzer et al., 1985; Ilgen et al., 2008; Witkiewitz et al., 2019) and it is difficult to predict with certainty who might do well over the long term with a moderated drinking strategy. Finally, in the current study, drinkers with high gratitude were more likely to have mild substance dependence; caution should be taken in making recommendations for moderated drinking to individuals with more severe dependence. Given these cautions, more research is needed to clarify optimal recommendations for treatment.

## Conclusion

A subset of individuals with alcohol use disorders who continue to drink also endorse high levels of gratitude at a 2.5-3 year follow up in a longitudinal study. These individuals at baseline exhibited a wide range of positive psychosocial factors and lower levels of clinical severity than their peers. The construct of trait gratitude might differ qualitatively between drinking non-AA members and sober AA members, warranting further research into the construct of trait gratitude and the context-specific ways that gratitude operates relative to 12-step programs.

## Figures and Tables

**Table 1.** Baseline differences between individuals diagnosed with alcohol dependence who reported higher than median gratitude and who were actively drinking (n=64) and their peers (n=211) in the Life Transitions Study.

Variable	High Gratitude Actively Drinking (n=64)	Others (n=211)
	M (SD) or % Yes	
*Demographics*		
Years of Education***	**15.5 (2.6)**	**14.2 (2.3)**
Age*	**40.9 (13.0)**	**45.3 (12.8)**
Income**	**$53.6k ($29.0k)**	**$42.0k ($27.3k)**
Employed**	**73.4% (n=47)**	**53.1% (n=112)**
Female*	**48.4% (n=31)**	**30.8% (n=65)**
Not European American	17.2% (n=11)	19.9% (n=42)
Married or Co-Habitating*	**50.0% (n=32)**	**35.1% (n=74)**
*Psychosocial Characteristics*		
Spirituality		
Daily Spiritual Experiences	54.5 (17.3)	54.0 (17.7)
Spiritual/Religious Practices	14.5 (6.9)	16.1 (7.7)
Positive Religious Coping	21.4 (8.0)	23.3 (7.8)
Negative Religious Coping***	**11.3 (2.4)**	**13.2 (4.0)**
Purpose in Life***	**102.7 (15.6)**	**89.7 (17.9)**
Forgiveness of Others	10.8 (2.9)	10.0 (3.3)
Forgiveness of Self***	**9.8 (3.8)**	**6.6 (3.6)**
Personality		
Neuroticism***	**20.8 (8.5)**	**26.2 (8.4)**
Extraversion***	**30.2 (6.1)**	**25.2 (8.1)**
Openness***	**32.1 (7.4)**	**28.1 (7.0)**
Agreeableness***	**34.0 (5.5)**	**30.5 (6.3)**
Conscientiousness***	**34.0 (6.9)**	**29.7 (7.1)**
*Clinical Characteristics*		
Mental health, addiction history, severity, recovery, baseline treatment type		
Want Abstinence as Outcome***	**37.5% (n=24)**	**78.2% (n=165)**
Had Previous Treatment**	**31.3% (n=20)**	**55.9% (n=118)**
Family History Alcohol Problems	87.5% (n=56)	87.6% (n=184)
Dependence Severity		
Severe***	**32.8% (n=21)**	**64.5% (n=136)**
Moderate	20.3% (n=13)	15.6% (n=33)
Mild***	**46.9% (n=30)**	**19.9% (n=42)**
Symptoms of physiological dependence	85.9% (n=55)	91.0% (n=192)
Alcoholics Anonymous Involvement***	**0.3 (0.7)**	**1.1 (1.5)**
Drinking Consequences***	**15.4 (9.7)**	**22.1 (11.6)**
Number of Psychiatric Symptoms**	**18.5 (11.3)**	**24.7 (13.0)**
Recent Negative Life Events*	**1.7 (1.8)**	**2.4 (1.8)**
Age at First Symptoms	26.8 (11.8)	29.5 (12.6)
Alcohol Use		
Percent Days Abstinent*	**47.4 (28.6)**	**58.8 (31.3)**
Drinks per Drinking Day***	**6.1 (5.0)**	**9.9 (8.1)**
Days Since Last Drink***	**13.0 (19.7)**	**28.8 (28.5)**
Percent Heavy Drinking Days	31.0 (28.5)	32.0 (29.4)
Treatment Type at Baseline		
Abstinence-based treatment***	**37.5% (n=24)**	**70.1% (n=148)**
Treatment permitting a moderated drinking goal***	**21.9% (n=14)**	**6.2% (n=13)**
Not in treatment at baseline***	**40.6% (n=26)**	**23.7% (n=50)**

* p<.05,

** p<.01,

*** p<.001.

Note: Chi Square analyses were inspected to determine statistical significance for Dependence Severity; asterisks indicate that adjusted residuals were > + or − 1.96, suggesting significant differences between groups of individuals for severe and mild dependence categories.

**Table 2.** Differences in Pearson correlations between trait gratitude and demographic, psychosocial, and alcohol use variables between non-AA members who are currently drinking and AA members sober 90 days or more

	Correlations with Trait Gratitude	
Variable	Actively Drinking Non AA Members (n=108)	AA Members with 90+ Days Abstinent (n=55)
*Demographics at Baseline*		
Years of Education*	**.223** *	**−.171**
Age	.000	−.061
Income*	**.213** *	**−.201**
*Psychosocial Characteristics*		
Spirituality		
Daily Spiritual Experiences	.428***^d^	.533***^c^
Spiritual/Religious Practices	.211*^d^	.098^c^
Positive Religious Coping	.263**^d^	.269*^c^
Negative Religious Coping	−.222*^d^	−.256^c^
Purpose in Life*	**.638** *** ^d^	**.371** ** ^b^
Forgiveness of Others	.296**	.337*
Forgiveness of Self	.353***	.367**
Personality		
Neuroticism	−.418***	−.374**
Extraversion	.369***	.296*
**Openness**^‡^	**.241** *	**−.052**
Agreeableness	.311**	.145
Conscientiousness	.337***	.223
*Clinical Characteristics*		
Alcoholics Anonymous Involvement	.073	.285*
Drinking Consequences	−.215*	−.151
Number of Psychiatric Symptoms	−.357***	−.309*
Recent Negative Life Events	.041^d^	−.150
Number of Previous Treatment Episodes	−.228*	−.038
Age at First Symptoms	−.100	−.085
Alcohol Use		
Percent Days Abstinent	.010	--^a^
Drinks per Drinking Day	−.258**	--^a^
Days Since Last Drink	.093	.320*
Percent Heavy Drinking Days	−.173	--^a^

‡ p<10,

* p<.05,

** p<.01,

*** p<.001.

Note. Symbols indicating statistical significance that appear in the “variable” column represent statistically significant differences between the correlation coefficients of the two groups. Symbols indicating statistical significance that appear in the “Non AA Members” and “AA Members” columns indicate statistically significant Pearson’s Correlations between trait gratitude and the variable in the first column for each group. Demographics, number of previous treatment episodes, and age at first symptoms were assessed at baseline, all of the other variables, including trait gratitude, were assessed at the final follow-up interview, 2.5 – 3 years after baseline.

a For individuals sober at least 90 days, Percent Days Abstinent was 100%, Drinks Per Drinking Day was 0, and Percent Heavy Drinking Days was 0% for all in this group, therefore, correlations could not be run.

b n=53,

c n=54,

d n=107.
